# The Relationship Between Obesity and Otitis Media with Effusion in Children

**DOI:** 10.22038/ijorl.2025.79859.3688

**Published:** 2025

**Authors:** Saleh Aghaei, Bijan Khademi, Mohammad Faramarzi, Amirhossein Babaei

**Affiliations:** *Otolaryngology Research Center, Department of Otolaryngology, Shiraz University of Medical Sciences, Shiraz, Iran.*

**Keywords:** Child, Otitis Media with Effusion, Obesity, Eustachian tube

## Abstract

**Introduction:**

Otitis media with effusion (OME) is a widespread condition affecting children globally. This study aimed to assess the relationship between obesity in pediatric populations and the risk of developing OME.

**Materials and Methods:**

This retrospective observational study was performed in 2020 at Khalili and Dastgheib hospitals, affiliated with Shiraz University of Medical Sciences in Shiraz, Iran. The study included all children aged 2 to 15 years with a confirmed OME diagnosis. Participants in the non-OME group were chosen from children who did not have OME.

**Results:**

A total of 148 healthy individuals were included in the non-OME group, while the OME group comprised 110 patients. Statistical analysis revealed that the mean age (p=0.040), weight (p<0.001), height (p=0.024), BMI (p=0.023), and BMI percentile (p=0.023) were significantly greater in the OME group compared to the non-OME group. Additionally, there was a higher proportion of males in the OME group (63.6%) compared to the non-OME group (44.0%), with this difference being statistically significant (p=0.001). Logistic regression analysis indicated that factors such as older age (p=0.023), male gender (p=0.001), and elevated BMI percentile (p=0.004) were significantly associated with the presence of OME.

**Conclusion:**

This research indicates that there is a correlation between obesity and a heightened risk of OME.

## Introduction

Otitis media with effusion (OME) is defined by fluid buildup in the middle ear without accompanying acute inflammatory signs, such as fever and pain (1). It is one of the most frequent diseases during infancy and childhood in both developing and developed countries (2).

OME is a leading contributor to conductive hearing loss in children. Research indicates that approximately 10% to 17% of children experience at least one episode of OME by the age of four (3). 

The frequency of OME has been increasing in the last decades, but the prevalence seems impossible to estimate due to the asymptomatic presentation of this condition (4). OME is usually associated with the Eustachian tube’s poor function, which leads to abnormal fluid drainage from the middle ear and causes middle ear effusion (5). 

Eustachian tube dysfunction may result from different reasons, including immaturity of the tube, adenoid inflammation, allergy, and congenital malformations (6). The association between OME and age, gender, socioeconomic status, and lifestyle has been demonstrated in several studies (7-9). In addition, obesity has been reported to be an important predisposing factor for developing OME (10-12).

Obesity poses a significant health challenge, elevating the likelihood of various disorders by altering the metabolism of adipose tissue, increasing fat accumulation, altering cytokine expression, the release of pro-inflammatory substances, and increasing gastroesophageal reflux (13,14). 

In children, obesity is defined as body mass index (BMI) equal to or more than 95 percentile (15). It seems that obesity and overweight may contribute to OME by increasing fat accumulation, gastroesophageal reflux diseases, and altering the pattern of cytokine expression (13). Some previous studies showed that children with higher BMI levels are more susceptible to developing OME (13,16,17). 

Conversely, a cohort study conducted by Venekamp et al. found no association between BMI at 6 and 11 months of age and the occurrence of otitis media during the first four years of life (18). Due to the controversy mentioned, we conducted this study to evaluate the prevalence of obesity in children diagnosed with OME.

## Material and Methods

This retrospective observational study was performed in 2020 at Khalili and Dastgheib hospitals, associated with the Shiraz University of Medical Sciences in Shiraz, Iran. These hospitals are major referral centers for ear, nose, and throat diseases in southern Iran.

The study included children aged 2 to 15 who had a confirmed diagnosis of OME, designating them as the OME group. 

The diagnosis of OME was made through clinical assessment, specifically by identifying an amber-colored tympanic membrane and a B- or C-type tympanogram. These evaluations were performed by a single academic otologist at the time of admission, utilizing otoscopic examination and impedance audiometry.

All patients received ventilation tube insertion following the guidelines for tympanostomy tube placement in children as outlined by the American Academy of Otolaryngology-Head and Neck Surgery (AAO-HNS) (19). 

The non-OME group was comprised of all children without OME who were referred to the hospitals for conditions unrelated to ear diseases.Patients with craniofacial anomalies, syndromic diseases, autoimmune disorders, immunodeficiency, infectious diseases, or malignancies were excluded from the study, as these conditions could interfere with the assessment of OME.

This research utilized a retrospective approach and a census design, including all individuals who satisfied the inclusion and exclusion criteria throughout the study. The study protocol and patient-informed consent documents received approval from the local Ethics Committee at Shiraz University of Medical Sciences (IR.SUMS.MED.REC.1398.656). 

Participation was voluntary, and the study's objectives and methods were thoroughly communicated to the parents or guardians, who provided written informed consent. Demographic and clinical features of patients recorded. 

Otoscopy and tympanometry were performed for all subjects in both groups. Weight was measured using digital scales (Seca: Hamburg, Germany), and heights were recorded with a portable stadiometer (Seca SMSSE-0260, Leicester, UK). The Body Mass Index (BMI) was determined by dividing an individual's weight in kilograms by the square of their height in meters (kg/m²) and recorded as such. Participants were grouped into four categories according to their BMI percentiles: underweight (BMI less than 5%), normal weight (BMI between 5% and 85%), overweight (BMI ranging from 85% to 95%), and obese (BMI of 95% or higher) (15).

Categorical variables were presented through frequencies and percentages, while quantitative variables were expressed as means accompanied by standard deviations (±SD). The Chi-square test was applied to assess potential relationships among categorical variables, and independent t-tests were used to analyse parametric continuous variables. 

Associations with a p-value below 0.2 were incorporated into the logistic regression analysis. A p-value below 0.05 was deemed statistically significant. All analyses were performed using SPSS version 25 (SPSS Inc., Chicago, IL, USA).

## Results

A total of 148 healthy subjects were recruited as the non-OME group, while the OME group comprised 110 patients diagnosed with OME. Among the 110 patients in the OME group, 13 (11.8%) reported a history of allergies. The demographic and anthropometric characteristics of both groups are presented in Table 1. The mean age of subjects in the OME group was significantly higher at 5.99 years (±2.80) compared to 5.19 years (±3.28) in the non-OME group (p=0.040), indicating that older age may be associated with higher likelihood of OME. Furthermore, there was a notable gender disparity: male participants constituted 63.6% of the OME group versus only 44.0% of the non-OME group, underscoring a significant difference (p=0.001). This finding suggests a predisposition towards OME in males relative to females.

**Table 1:** Demographic data and anthropometric measurements in both groups.

**Variable**		**Group**		**p-value**
		**OME (N=110)**	**Non-OME (N=148)**	
Age, mean (±SD)		5.99 (2.80)	5.19 (3.28)	0.040^
Gender	Male, N (%)	70 (63.6%)	66 (44.0%)	0.002*
	Female, N (%)	40 (36.4%)	84 (56.0%)	
Weight, mean (SD)		23.77 (10.76)	18.47 (8.19)	<0.001^
Height, mean (SD)		115.34 (18.42)	110.71 (12.54)	0.024^
BMI, mean (SD)		17.14 (4.09)	14.51 (3.29)	<0.001^
BMI percentile, mean (SD)		54.04 (38.77)	23.45 (30.32)	<0.001^
BMI category	Underweight, N (%)	22 (20.0%)	73 (48.7%)	<0.001*
	Normal, N (%)	49 (44.5%)	65 (43.3%)	
	Overweight, N (%)	17 (15.5%)	9 (6.0%)	
	Obese, N (%)	22 (20.0%)	3 (2.0%)	

*Chi-square test ^ Independent sample t-test

**Fig 1:**
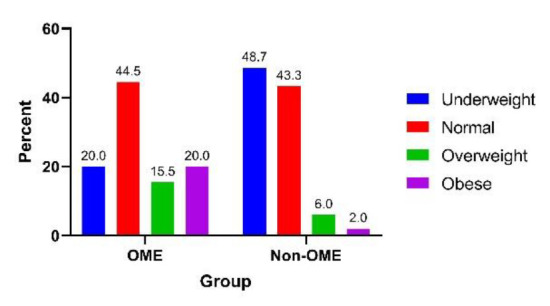
The frequency of each BMI category in OME and non-OME groups.

Anthropometric measurements revealed that the mean weight among patients with OME was substantially higher at 23.77 kg (±10.76) compared to 18.47 kg (±8.19) in the non-OME group, with a p-value of <0.001. Similarly, the OME group exhibited significantly greater mean height (115.34 cm, ±18.42 vs. 110.71 cm, ±12.54, p=0.024) and BMI (17.14, ±4.09 vs. 14.51, ±3.29, p<0.001). The BMI percentiles further illustrated a stark contrast, averaging 54.04 (±38.77) in the OME group as compared to 23.45 (±30.32) in the non-OME group (p<0.001). The distribution of body categories in terms of BMI classification was notably different between the groups. In the OME group, 20.0% of patients were classified as underweight, while a significant 20.0% were obese. Conversely, the non-OME group presented a higher prevalence of underweight individuals (48.7%) and significantly fewer obese individuals (2.0%), as demonstrated in Figure 1.

A logistic regression analysis was conducted to explore further the associations between various factors and OME (Table 2). The results indicated that age was positively correlated with OME, with an odds ratio of 1.065 (p=0.210), suggesting that for each additional year in age, the likelihood of having OME increased slightly, although this was not statistically significant. Male gender is strongly associated with OME, indicated by an odds ratio of 2.743 (p=0.001), implying that males were significantly more likely to develop OME than females when controlling for other factors.

**Table 2:** Logistic regression test for factors associated with OME

**Variables**	**Beta coefficient**	**Standard error**	**Wald test**	**Degree of freedom**	**p-value**	**Odds ratio**	**95% confidence interval for the odds ratio**	
							**Lower**	**Upper**
Age	0.063	0.050	1.572	1	0.210	1.065	0.965	1.175
Gender	1.009	0.291	12.005	1	0.001	2.743	1.550	4.853
BMI percentile	-0.027	0.004	37.579	1	<0.001	0.973	0.965	0.982

Furthermore, a higher BMI percentile emerged as a significant factor associated with OME, showcasing an odds ratio of 0.973 (p<0.001). This result implies that the odds of having OME marginally decreased for each incremental increase in BMI percentile.

## Discussion

In this study, we discovered a link between obesity and OME in children. Our findings indicated that children with OME had notably higher BMI levels than those without the condition. Increased fat accumulation, gastroesophageal reflux diseases, and altered cytokine expression patterns could explain it.

Some previous studies suggested a relationship between obesity and OME (13, 16, 17, 20). In a study by Kaya et al., the association between pediatric obesity and OME was assessed. The BMI level in patients with OME was significantly higher than the BMI of healthy children (p-value=0.044) (21). A study by Haksever et al. in 2022 found that the BMI is significantly higher in the OME than in the control group (10). Kim et al. examined the link between childhood obesity and BMI in a study involving 140 children who underwent unilateral or bilateral ventilation tube insertion for the treatment of OME and 190 healthy children. Their findings revealed a significantly higher prevalence of obesity in the experimental group compared to the control group, leading them to propose that obesity may lead to the development of OME (22). The findings of a prospective cohort study showed that obese children are about 2 times more susceptible to OME. The study also indicated that socioeconomic factors, breastfeeding history, and the presence of allergic or chronic adenoid/tonsil disorders did not influence the relationship between obesity and OME (23). Furthermore, a retrospective cohort study conducted in 2020 revealed that children who had ventilation tube insertion were at a higher risk of being overweight when compared to their peers matched for age and gender (24).  Additionally, two recent studies indicated a greater prevalence of overweight and obesity in children diagnosed with OME (13, 21). In the study by Alaraifi et al., the average BMI of the children in the study group was notably higher than that of the control group, with a significance level of P=0.032. Furthermore, this study revealed that obese children with OME have an increased likelihood of experiencing recurrent episodes of OME (17). Consistent with the aforementioned studies, our results are similar to those of previous studies, and we showed a significant difference between BMI in the OME and non-OME groups. On the contrary, some studies found no relation between obesity and OME. Choi et al. conducted a study examining the dietary intake differences between children aged 4 to 13 years with and without OME. Their research revealed that elements like overall calorie consumption, BMI category, hydration levels, protein intake, carbohydrate distribution, and sodium intake showed no significant association with the occurrence of OME (16). An investigation by Venekamp et al. (2016) revealed no significant relationship between BMI at 6 and 11 months of age and the occurrence of otitis media in the first four years of life. One major drawback of their study was the dependence on weight and height data collected at 6 months to evaluate early-life BMI. (18). In our study, the relation between age and OME was insignificant based on logistic regression.

Our research found a higher prevalence among males (63.6%) compared to females (36.4%). Similarly, Parmar et al. (25) reported a greater prevalence in males (58.97%) and a lesser prevalence in females (41.03%). Additionally, Sharma et al. (26) found a prevalence of 62% in males and 38% in females. Consistent with these findings, our study also identified a higher prevalence among males than females.

A key strength of our study is its relatively large sample size. However, a notable limitation is that our data collection did not encompass a broader range of demographic characteristics and other potential risk factors for OME, including socioeconomic status and lifestyle factors.

Further studies with larger sample sizes and more comprehensive biochemical investigations are required to clarify this association.

## Conclusion

 In summary, our research suggests that there may be a link between pediatric obesity and the OME. Physicians should be aware of this issue, and precise evaluation of obese children is necessary.

## References

[1] Otteson T (2022). Otitis Media and Tympanostomy Tubes. Pediatr Clin North Am..

[2] Schilder AG, Chonmaitree T, Cripps AW, Rosenfeld RM, Casselbrant ML, Haggard MP (2016). Otitis media. Nat Rev Dis Primers.

[3] Towerman AS, Hayashi SS, Hayashi RJ, Hulbert ML (2019). Prevalence and nature of hearing loss in a cohort of children with sickle cell disease. Pediatr Blood Cancer.

[4] Vanneste P, Page C (2019). Otitis media with effusion in children: Pathophysiology, diagnosis, and treatment A review. J Otol.

[5] Searight FT, Singh R, Peterson DC ( 2022). Otitis Media With Effusion. StatPearls.

[6] Goulioumis AK, Gkorpa M, Athanasopoulos M, Athanasopoulos I, Gyftopoulos K (2022). The Eustachian Tube Dysfunction in Children: Anatomical Considerations and Current Trends in Invasive Therapeutic Approaches. Cureus.

[7] Byeon H (2019). The association between allergic rhinitis and otitis media: A national representative sample of in South Korean children. Sci Rep.

[8] Songu M, Islek A, Imre A, Aslan H, Aladag I, Pinar E (2020). Risk factors for otitis media with effusion in children with adenoid hypertrophy. Acta Otorhinolaryngol Ital.

[9] Korona-Glowniak I, Wisniewska A, Juda M, Kielbik K, Niedzielska G, Malm A (2020). Bacterial aetiology of chronic otitis media with effusion in children - risk factors. J Otolaryngol Head Neck Surg.

[10] Haksever M, Durgut O, Demirci H, Gençay S, Özmen S (2022). Relationship between otitis media with effusions and pediatric obesity. Int J Pediatr Otorhinolaryngol..

[11] Cao J, Liu W, Yang Z, Qu G, Zhong C (2024). Causal Relationship Between Body Mass Index and Risk of Otitis Media with Effusion in Children: A Mendelian Randomization Study. Indian J Otolaryngol Head Neck Surg.

[12] Yang A, Jv M, Zhang J, Hu Y, Mi J, Hong H (2023). Analysis of Risk Factors for Otitis Media with Effusion in Children with Adenoid Hypertrophy. Risk Manag Healthc Policy..

[13] Gavrilovici C, Spoială EL, Ivanov AV, Mocanu A, Ștreangă V, Alecsa MS (2021). Otitis Media and Obesity-An Unusual Relationship in Children. Healthcare (Basel).

[14] Krajewska Wojciechowska J, Krajewski W, Zatoński T (2019). The Association Between ENT Diseases and Obesity in Pediatric Population: A Systemic Review of Current Knowledge. Ear Nose Throat J.

[15] Peinado Fabregat MI, Saynina O, Sanders LM (2023). Obesity and Overweight Among Children With Medical Complexity. Pediatrics.

[16] Choi HG, Sim S, Kim SY, Lee HJ (2015). A high-fat diet is associated with otitis media with effusion. Int J Pediatr Otorhinolaryngol.

[17] Alaraifi AK, Alosfoor MA, Alsaab F (2020). Impact of pediatric obesity on the prevalence and outcome of otitis media with effusion. Int J Pediatr Otorhinolaryngol..

[18] Venekamp RP, Menger JT, Uiterwaal CS, van der Ent CK, Smit HA, Schilder AG (2016). Lack of Impact of Body Mass Index at Young Age on Otitis Media Occurrence During Preschool Years: Wheezing Illnesses Study Leidsche Rijn Cohort Study. Pediatr Infect Dis J.

[19] Rosenfeld RM, Schwartz SR, Pynnonen MA, Tunkel DE, Hussey HM, Fichera JS (2013). Clinical practice guideline: Tympanostomy tubes in children. Otolaryngol Head Neck Surg.

[20] Omer CA, Shem AAM (2022). Association between otitis media with effusion & body mass index in preschool-age children. AMJ (Advanced Medical Journal) is the scientific journal of Kurdistan Higher Council of Medical Specialties.

[21] Kaya S, Selimoğlu E, Cureoğlu S, Selimoğlu MA (2017). Relationship between chronic otitis media with effusion and overweight or obesity in children. J Laryngol Otol.

[22] Kim SH, Park DC, Byun JY, Park MS, Cha CI, Yeo SG (2011). The relationship between overweight and otitis media with effusion in children. Int J Obes (Lond).

[23] Kuhle S, Kirk SF, Ohinmaa A, Urschitz MS, Veugelers PJ (2012). The association between childhood overweight and obesity and otitis media. Pediatr Obes.

[24] Klein A, Kraus O, Luria A, Ovnat Tamir S, Marom T (2020). Are Children Scheduled for Ventilation Tubes Insertion Overweight? A Cohort of Israeli Children. Ann Otol Rhinol Laryngol.

[25] Parmar S, Davessar JL, Singh G, Arora N, Kansal L, Singh J (Nov 2019). Prevalence of Otitis Media with Effusion in Children with Hearing Loss. Indian J Otolaryngol Head Neck Surg.

[26] Sharma K, Mehan R, Arora A (2015). Clinico-audio-radiological and operative evaluation of otitis media with effusion. Indian Journal of Otology.

